# Innovative Method of Using Endoscope in Postoperative Canal Wall Down Mastoid Cavity

**Published:** 2018-06-30

**Authors:** Meera Bista, Nayan Bahadur Mahato, Deepak Regmi

**Affiliations:** 1 Department of ENT-HNS, Kathmandu Medical College, Sinamangal, Kathmandu, Nepal

**Keywords:** *canal wall down mastoidectomy*, *cleaning of mastoid cavity*, *oto-endoscopy*, *recidivism*

## Abstract

**Introduction:**

Poor access to the difficult areas in the middle ear and mastoid cavity is considered as the major reason for failure in mastoid surgery. Wide field visibility, visualization of nooks and corners by an endoscope could contribute to better clinical control of the disease in these patients that cannot be accessed by the operating microscope. The study was done to assess and clean postoperative canal wall down mastoidectomy cavities with endoscope and compare with oto-microscopy.

**Methods:**

This was a descriptive cross-sectional study, done in Kathmandu Medical College from January to June 2017. Thirty two patients were included in the study. Data collection was done by convenient sampling. Statistical analysis was done by Chi square test and Fisher Exact test, P value of <0.005 was considered statistically significant.

**Results:**

The study revealed that exposure benefit with an endoscope in canal wall down mastoid surgery was significantly better than with a microscope (P value of 0.034). The level of complete clearance and level of difficulty in cleaning with the help of a microscope compared to endoscope did not show a significant difference with P value of 0.288 and 0.652 obtained by Fisher extract test respectively. After microscopic removal of materials from the mastoid cavity, 22 (68.8%) which is more than half of cases had remaining materials in the cavity which was removed by endoscope completely.

**Conclusions:**

Outcome will make the ENT surgeons aware of use of endoscopy in post mastoid follow up cases to give better results and make the surgeon much more successful in his/her endeavor to eradicate the disease.

## INTRODUCTION

Modified radical mastoidectomy is a well established treatment in chronic suppurative otitis media atticoantral type. Success of the surgery depends on complete eradication of the disease from the middle ear cleft. Poor access to the difficult areas in the middle ear and mastoid cavity is considered as the major reason for failure in mastoid surgery^1^ which can lead to a chronically discharging cavity, frustrating the patient as well as the operating surgeon.

Thomassin and colleagues in 1987 in France devised the first endoscopically guided otosurgery in the prevention of residual cholesteatoma. This landmark article showed a considerable reduction in residual cholesteatoma attributed to the advent of endoscopic evaluation of blind spots encountered during the primary surgery.^2^ Inspite of various technical advancements in operating microscope, basic limitations could not be resolved.^3^

Wide view provided by the endoscope enables a more comprehensive examination of mastoid cavity and visualization of areas that cannot be assessed by the operating microsope.^4^

## METHODS

This was a descriptive cross-sectional study conducted in Kathmandu Medical College (KMC), Sinamangal from January 2017 to December 2017. A total of thirty -two patients were included in the study. Data collection was done by convenient sampling and single blinded technique was used for exploration of mastoid cavity to avoid experimental bias. Cases were selected from patients who visited department of Ear Nose Throat and Head and Neck Surgery (ENT-HN Surgery), KMC receiving periodic follow-up for canal wall down mastoid surgery in the study duration. Patients who consented for the study from age group 10 to 70 years of both sexes who had history of canal wall down mastoidectomy performed at least 60 days before enrollment were included. Those patients who refused enrollment, age less than 10 and more than 70 years or presence of surgical complications were excluded from the study. Ethical approval was obtained from Institutional Review Committee of Kathmandu Medical College and consent of the patient was taken for the research work.

Patients fulfilling the inclusion criteria were first examined under microscope (f = 250mm) in lying position and cleaning of debris as well as evaluation of cavity was done. Regarding the amount of material requiring removal, semi quantitative scale was used: large amount = over 50% of cavity filled with material; moderate= 25 to 50% of cavity filled with material; small= less than 25% of cavity filled with material. A set of questionnaire was given to the researcher to fill in.^5^ Again the same patient was evaluated under endoscope in same position by the principle investigator. Zero degree & 30 degree 4mm wide 10 cm long Hopkins rod endoscopes were used. Any debris or secretion left behind was removed and reevaluation of cavity was done. All endoscopic cleaning were performed by direct visualization on the monitor. Forceps and suction tips were those used routinely for ear microscopic procedure. Again a set of questionnaire was given to the principal investigator to answer. Then qualitative analysis of the finding was performed.

The collected data was managed by SPSS 16 and biostatistician's help was taken during data analysis.

## RESULTS

**Figure 1.**
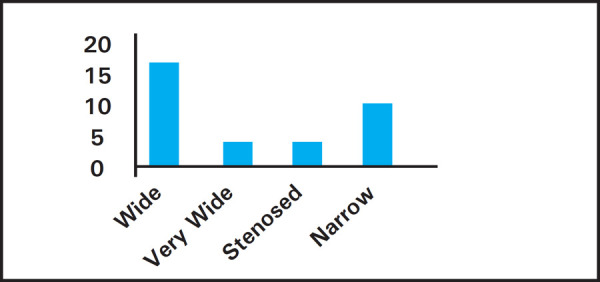
Results of meatoplasty.

The study comprised of 32 patients. Age ranged between 10 to 53 years (mean 24years). Laterality showed 22 right ear involvement and 10 left ear involvement. Three cases had stenosis of meatus 3 (9.3%) which led to difficulty in cleaning under microscope (Figure 1).

Only one case had recidivism (3.12%) with foul smelling aural discharge and graft perforation. Level of difficulty in cleaning the crusts, secretions and debris from the mastoid cavity by microscopic method and endoscopic method did not show significant difference with value of 0.652 by Fisher Extract test (Table 1).

**Table 1.** Level of difficulty in cleaning (level micro x level endo).

	level endo				Total
		1	2	3	
	1	1	1	5	7
level micro	2	2	2	9	13
	3	1	0	11	12
Total		4	3	25	32

levelmicro = level of difficulty in cleaning by microscopy

levelendo = level of difficulty in cleaning by endoscopy

Moderate = 1

Major = 2

Minor = 3

The exposure benefit with a microscope was much less than with an endoscope revealing a significant P value of 0.034 (Table 2).

**Table 2.** Exposure benefits by microscopic and endoscopic method (area of visibility x area of visibility).

		area of visibility	Total
area of visibility	complete	10	10
	partial	22	22
Total		32	32

The level of cleaning with the help of a microscope compared to endoscope did not show a significant difference with P value of 0.288 obtained by Fisher extract test

**Table 3.** Level of completeness of cavity cleaning (Level of cleaning).

		Cleaning Complete	Partial	Total
level of cleaning	Complete	9	0	9
	Partial	18	5	23
Total		27	5	32

With the help of microscope complete cleaning was 9 (28.1%) whereas with endoscope it was 27 (84.4%), showing much better result with endoscope (Table 4).

**Table 4.** Compression of complete cleaning (Level of cleaning).

		Cleaning Complete	Partial	Total	
level of cleaning		Count	9	0	9
	complete	% of Total	28.1	0.0	28.1
		Count	18	5	23
	partial	% of Total	56.2	15.6	71.9
	Total	Count	27	5	32
% of Total		84.4%	15.6	100	

After removing the materials from the mastoid cavity by microscopic method 22 (68.8%) which is more than half of cases had remaining materials in the cavity which was removed by endoscope completely (Table 5).

**Table 5.** Materials left in cavity after microscopic cleaning (Any discharge left).

		n (%)	Valid %	Cumulative Percent
	No	10 (31.3)	31.3	31.3
Valid	yes	22 (68.8)	68.8	100.0
	Total	32 (100)	100.0	

The common sites for collection of crusts, discharge and debris left behind were anterior attic area and sinodural angle followed by retrofacial area. Mastoid antrum and tip cell area showed crusts and debris especially in cases where high facial ridge was seen. These were the key areas which were completely cleared by direct vision using an endoscope which had the propensity of developing into ricidivism.

## DISCUSSION

Chronic suppurative otitis media atticoantral type is considered a dangerous disease due to its potentiality to cause lethal complications. Canal wall down mastoidectomy with reconstruction of hearing mechanism and adequate meatoplasty is one of the chosen methods for treating such a condition. The operation aims to make the diseased ear safe and dry. Chronically discharging cavity is a threat to the patient and a challenge for the operating surgeon. One of the most important causes for such a scenario is inadequate cleaning of crusts, debris and secretions from the post operated cavity. This in turn is due to inadequate visualization by a microscope which has a straight line vision and cannot see the nooks and corners where diseases hide themselves.

The result of the study showed that significant benefit is obtained on visibility of operated canal wall down (CWD) mastoid cavity by using endoscope. In 68.8% of cases explored under microscope revealed material left in the cavity which was completely removed by using an endoscope. This was because of proper visualization of difficult areas not seen by a microscope. These areas were anterior attic region, sinodural angle and retrofacial region respectively. Mastoid antrum and tip cells were the areas not properly coming to vision under microscope where facial ridge was not properly lowered. Similar results were revealed in a study done by Freire GSM et al where he showed endoscopy proved benefit in 61.1% of cases, so in more than half cases endoscope was able to expose areas not visualized under microscopy. Comparison of results between microscopic and endoscopic evaluation showed significant difference (P<0.0002).^5^ Khalil HS in his study 'Canal wall down mastoidectomy: A long term commitment to the outpatients?' Concluded that the decision of performing CWD mastoidectomy in Atticoantral disease is not to be undertaken lightly as the patient will become a regular visitor to the outpatient for many years to come.^6^ An old epigram states, 'Once you have operated on 1000 ears, you need never see another patient'. In the case of cholesteatoma surgery these words convey a sad but at present inevitable truth.

In the 1990s, as an extension of these anatomic studies, investigators examined the application of endoscopes as observational tools in cholesteatoma second-look procedures to evaluate their ability to detect residual or recurrent disease.^7–11^

One case in the study showed recidivism in the form of recurrence with foul smelling discharge and graft perforation as well as retraction. Baharawy et al in his study named 'Evaluation of endoscopic surgery for middle ear cholesteatoma' stated that recidivism is the main problem after cholesteatoma surgery. It means detection of cholesteatoma matrix during planned second look or unplanned revision. ^12^ Recidivistic cholesteatoma may be either residual or recurrent form.^13,14^ Residual cholesteatoma is due to inadequate primary resection whereas recurrence is due to new cholesteatoma formation after successful disease removal. Several reports have presented the utility of endoscopic ear surgery as a method for lowering the rates of residual and recurrent disease after cholesteatoma removal. ^15^ The drawback of endoscope is its heat generation after long application, one hand procedure, fogging and the learning curve.

The limitation of this study is its small number of cases and larger studies are needed in the future. CONCLUSIONS

Endoscopes for ear surgeries are a very useful armamentarium in the basket of ENT surgeons. Poor access to the difficult areas in the middle ear and mastoid cavity is considered as the major reason for failure in mastoid surgery. Application of ear endoscopy in post mastoidectomy cavity to see the persistence of debris can facilitate better removal. Wide field visibility, visualization of nooks and corners could contribute to better clinical control of the disease in these patients which make the surgeon much more successful in his/ her endeavor to eradicate the disease.
